# Association between high sensitivity C-reactive protein (hs-CRP) levels and the risk of major adverse cardiovascular events (MACE) and/or microembolic signals after carotid angioplasty and stenting

**DOI:** 10.22088/cjim.10.4.388

**Published:** 2019

**Authors:** Farzaneh Foroughinia, Amir Ashkan Tabibi, Haniyeh Javanmardi, Anahid Safari, Afshin Borhani-Haghighi

**Affiliations:** 1 Clinical Neurology Research Center, Shiraz University of Medical Sciences, Shiraz, Iran; 2 Department of Clinical Pharmacy, School of Pharmacy, Shiraz University of Medical Sciences, Shiraz, Iran; 3 Student Research Committee, Shiraz University of Medical Sciences, Shiraz, Iran; 4 Stem Cells Technology Research Center, Shiraz University of Medical Sciences, Shiraz, Iran; 5 Department of Neurology, School of Medicine, Shiraz University of Medical Sciences, Shiraz, Iran

**Keywords:** Stroke, Carotid, Angiography, Angioplasty, Inflammation, C-reactive protein, Complication

## Abstract

**Background:**

To evaluate the association between pre/post-procedural high sensitive C-reactive protein (hs-CRP) level and hs-CRP difference, and the risk of major adverse cardiovascular events (MACE) or new diffusion-weighted MRI lesions after carotid angioplasty and stenting (CAS).

**Methods:**

In this study, conducted in 2016 in Shiraz (Iran), patients who underwent diagnostic angiography and CAS were recruited. CAS was performed with distal embolic protection device on patients with both standard and high risk of endarterectomy. Pre/post-procedural hs-CRP, and hs-CRP difference were determined by immunoenzymometric assay method.

**Results:**

A total of 50 patients with diagnostic angiography and 60 patients with CAS were enrolled. No death, myocardial infarction, ischemic or hemorrhagic stroke, and need to revascularization occurred during the 30-days of the post-procedural period. Accordingly, the statistical evaluation in associating MACE and hs-CRP levels was impossible. Angioplasty was associated with higher frequency of elevated post-procedural hs-CRP in comparison to angiography (P=0.003). The higher age, symptomatic lesions, negative history of hypertension, and hs-CRP difference had significant association with the presence of new DWI lesions in univariate analysis (all P<0.05). Angioplasty of left carotid bulb and post-procedural hs-CRP levels was very close to the level of significance (P=0.06). But only left sided lesions had positive association (P=0.037) and hypertension had negative association (P=0.037) in multivariate regression analysis. There were significant association between post-procedural hs-CRP level (P=0.02) and hs-CRP difference (P=0.003), and the number of new lesions; and the hs-CRP difference and the accumulated lesion surface area (P=0.009).

**Conclusion:**

Post-procedural hs-CRP and hs-CRP difference may predict embolic complications of CAS.

Stroke is the first cause of morbidity and the third cause of mortality around the world (1). Large arterial stenosis is a treatable cause of ischemic stroke (2). Although carotid artery angiography and carotid angioplasty and stenting (CAS) are good diagnostic and therapeutic options in patients with severe carotid stenosis and occlusion (3, 4). Ischemic strokes, myocardial infarction and death are the most serious complications of these procedures (5).

CAS procedural success can be evaluated by a composite outcome of death, stroke, myocardial infarction (MI), or repeat revascularization of the target lesion similar to major adverse cardiovascular events (MACE) for coronary interventions (6). Microembolic brain infarcts represent emboli, which may be dislodged during a procedure (7). Although the rate of symptomatic presentation of these infarcts ranges 2%-17%, their harmful effects on cognition are well-established (6, 8). Hence, reducing MACE and the rate of microembolic signals is the most important aim in any CAS modification. C-reactive protein (CRP) is an important marker of systemic inflammation that is produced in the liver in response to pro-inflammatory cytokines (9). Serum CRP measurement, using high-sensitivity assay techniques (hs-CRP) enables the diagnosis of vascular inflammation even in the subclinical state (10). Furthermore, CRP also induces the release of monocyte tissue factor, contribute to plaque rupture and acute thrombotic events such as myocardial infarction and stroke (11, 12). 

Many studies have discussed the relation between hs-CRP level and the occurrence of atherosclerotic and thrombotic events (MI and stroke) (-). Elevated baseline CRP is a powerful predictor for myocardial necrosis in patients undergoing coronary stenting (16, 17). This is a prospective study to evaluate whether pre-procedural hs-CRP, post-procedural hs-CRP, and/or post-pre-procedural difference in hs-CRP level (*hs-CRP difference*) is associated with the risk of MACE or microembolic signals in patients undergoing carotid artery angioplasty and stenting.

## Methods

***Study population, demographic variables and ethics: *** This cohort study was conducted in September to December 2016 at Kowsar Hospital, a high-volume referral center for CAS, affiliated with Shiraz University of Medical Sciences, Shiraz, Iran. Successive patients with carotid artery stenosis were included in this study. Diagnostic workup such as Doppler sonography of intracranial and extracranial arteries, CT and/or MR angiography and laboratory investigations were performed for all patients. Those with positive history of active intracranial hemorrhage, cerebral infarction caused by cardio-aortic emboli, lacunar stroke, vasculitis, arterial dissection, fibromuscular dysplasia, and previous disabling stroke (with modified Rankin Scale score of ≥3) were excluded. Patients who had contraindications of angiography such as coagulopathy, previous allergic reactions to contrast media, renal failure, kidney diseases and fever with unknown cause were excluded from the study. Patient's demographic variables such as age and gender were recorded. Patients were also evaluated for major atherosclerotic risk factors such as current or previous smoking, dyslipidemia (positive history and/or fasting cholesterol level >200 mg/dl or fasting triglyceride level >180 mg/dl, or low density lipoprotein>130 mg/dl), arterial hypertension (positive history and/or systolic blood pressure ≥140 mm Hg, diastolic blood pressure ≥90 mm Hg, treated or not treated), and any history of diabetes mellitus (positive history and/or FBS ≥ 126 mg/dl or 2-hpp blood glucose ≥ 200 mg/dl). This study was approved by the local Ethics Committee of Shiraz University of Medical Sciences, Shiraz, Iran (No# 12616). A written informed consent was obtained from each patient before recruitment. 

***Angiography and carotid stenting protocols: ***Cerebral angiography and CAS were performed according to standardized protocol, as previously described in detail (4). Patients with stenosis of more than 50% in noninvasive procedures underwent angiography to determine their treatment path. Symptomatic patients with more than 50% stenosis and asymptomatic patients with greater than 70% were recruited for stenting procedure according to NASCET criteria (18). Both standard and high risk patients for endarterectomy were included. All patients received antiplatelet therapy, clopidogrel (plavix) 75 mg daily with a loading dose of 600 mg, and aspirin 80 mg daily after a loading dose of 325 mg at least 48 hours (preferably four days) prior to the procedures. Laboratory methods to evaluate the resistance to anti-platelet drugs were unavailable in our center. Both angiography and CAS procedures were done under local anesthesia. Distal embolic protection device (EPD) was used for all patients in CAS group. Closed cell [Wallstent (Boston Scientific, Natick, Mass., USA)], or hybrid stent [Cristallo Ideale (Invatec Technology, Frauenfeld, Switzerland)] self-expanding stents (6-8mm diameter) was placed across the stenotic segment. Pre- and/or post-dilation was performed for those who required it. Patients were discharged one day after the procedure by a neurologist and were prescribed 75 mg of clopidogrel daily for twelve months and 80 mg of aspirin for the rest of their lives. 

***Blood sampling procedures: ***CRPs were measured using highly-sensitive enzyme-linked immunosorbent assay (ELISA) kit (Monobind INC, Lake forest, CA 92630, USA) with immunoenzymometric assay method. Twenty-four hours before and after the procedure, 5 ml fasting venous blood sample was collected from each patient. We calculated the hs-CRP level by using a standardized curve in kit catalogue. The cutoff value for the elevated hs-CRP was set at 3 mg/L. 

***Post-procedural MRI technique: ***Post-procedural MRI was performed within the first 24 hours after the stenting procedure using Siemens MR 1.5 Tesla machine. MRI sequences included axial diffusion-weighted imaging (DWI), and apparent diffusion coefficient (ADC) (TR/ TE: 100/ 1000, flip angle: 90, 5.5-6 mm slice thickness). Maximum spatial gradient magnetic field value of 3.3 Tesla/meter or less, and a maximum whole body averaged specific absorption rate (SAR wb) of 2.0W/kg for 15 minutes of MR scanning were considered for all procedures. To determine if a new ischemic lesion has occurred during the study and differs them from the old ones, only diffusion-restricted lesions (hyper-intense in DWI and hypo-intense in ADC) were recognized as new lesion. Presence or absence of new lesions, number, total accumulative surface area, and average surface area of the lesions (accumulated lesional surface area divided into number of lesions) were recorded.

***Follow-up: ***All patients in both groups were re-examined in a regular time interval (immediately after the procedure and on days 1 and 30) for ischemic or hemorrhagic stroke, myocardial infarction, need for revascularization, or any other post procedural complications. MACE was reported by a single expert neurologist who was blinded to the independent variables.

***Statistical analysis: ***Data were analyzed using SPSS Version 16.0 (SPSS Inc., Chicago, Ill., USA).Categorical values are presented as counts and percentages and clinical variables are expressed as means ± standard deviations. In addition, t-test and Mann Whitney test were used to compare hs-CRP level between groups. Chi-square test was performed to determine the association between qualitative variables and outcomes. 

The correlation between hs-CRP serum level and the number of new lesions was determined by Kendall's test. A P value (P<0.05) was considered to be statistically significant. Univariate logistic regression analysis was done to evaluate the association between the clinical and procedural variables and hs-CRP levels, and development of new DWI lesions. Due to clinical significance of some variables, multivariate logistic regression was performed for factors in association with new DWI lesions with P<0.1. 

## Results

**Angiography and CAS group: **A total of 50 patients who underwent diagnostic angiography and 60 patients who underwent CAS were included in this study. Thirty-three (66%) of patients in the angiography group and 41 (68%) in the CAS group were males. The mean ages were 62.7+10.71 [95%confidence interval (CI): 59.74-65.52] in angiography group and 70.68+8.09 (CI: 68.61-72.61) for CAS group. Table-1 shows the demographic, clinical characteristics and percentiles of pre/post procedural hs-CRP in angiography and angioplasty groups.

**Table 1:** Demographic characteristics, associated diseases, and number and percentages of elevated hs-CRP

**Variable**	**angiography group** **N=50**	**CAS group** **N=60**	**P value**	**Effect size**
Mean age (years)	62.7+10.7	70.7+ 8.09	< 0.001	-1.17
Gender , male %	33 (66%)	41 (68%)	0.795	0.025
Weight	67.90±11.9	68.82±10.7	0.67	-0.011
Diabetes mellitus	16 (32%)	19 (31.7%)	0.970	0.004
Hypertension	42 (84%)	52(87%)	0.693	0.038
Hyperlipidemia	29 (58%)	41 (68%)	0.262	0.106
Smoking	8 (16%)	14 (23%)	0.338	0.091
Previous TIA^1^ or stroke	27(54%)	40 (66.6%)	0.175	0.128
Previous IHD^2^	15 (30%)	29 (48.3%)	0.051	0.183
Elevated pre-procedural hs-CRP	14 (28%)	20 (33.3%)	0.547	0.057
Elevated post-procedural hs-CRP	21 (42%)	39 (65%)	0.016	0.224

1:TIA: transient ischemic attack 2:IHD: Ischemic heart disease

The angiography/plasty procedure was well endured by all patients. The mean stenosis of visualized carotid arteries in angiography group was 31.4±11.25 (CI: 27.91-34.09), and the mean stenosis of operated carotid arteries in CAS group was 81.05±13.50% (CI: 77.72-84.77). A total of 34 left internal carotid arteries and 26 right carotid arteries were stented. Embolic protection device (EPD) was used for all patients in the CAS group. The closed cell stent (Wallstent) was deployed in 41 of the participants, and the hybrid stent (Cristallo) was deployed in 19 patients. In the angiography group, mean pre-procedural hs-CRP was 4.39±9.92 mg/ml (CI: 1.98-7.33). In the angioplasty group, mean pre-procedural hs-CRP was 2.88±4.05 mg/ml (95%CI: 1.94-3.98). The post-procedural hs-CRP in angiography and angioplasty groups were 9.07±24.90 mg/ml (95%CI: 2.94-16.69), and 7.26±14.25 mg/ml (CI: 4.43-11.12), respectively. Mean hs-CRP difference was 4.67±17.51mg/ml (95%CI: 0.46-10.38) in angiography group, and 4.31±13.08 mg/ml (95%CI: 2.02-8.06) in patients underwent CAS. Hs-CRP difference in CAS group was significantly higher in comparison with angiography group (P=0.012). Table-1 showed the percentile of elevated post-procdural hs-CRP was significantly higher in angioplasty group in comparison to angiography group.

**MACE, microembolic signals and hs-CRP concentration in CAS group: **No death, myocardial infarction, ischemic or hemorrhagic stroke, need of revascularization occurred during the 30-day post-procedural period. Table-2 shows the association between the demographic parameters, risk factors, anatomical and procedural variables, plus hs-CRP levels with the presence of new lesion in CAS group. The correlation between the hs-CRP difference and developing new lesions reached a statistically significant level. (P=0.01) Hs-CRP difference had a significant association with the number of new lesions (R=0.29, P=0.003) and the accumulated lesion surface. (R=0.38, P=0.009). There was also a significant association between the post-procedural hs-CRP level and the number of ischemic lesions (R=0.22, P=0.02). However, the correlation between the post-procedural hs-CRP and total lesion area did not reach a significant level (R=0.22, P=0.13).

**Table 2:** Association of demographic variables, risk factors, anatomical/procedural variations and hs-CRP levels with developing new lesion in patients underwent CAS

variables		**New lesion**		**p-value**	**Effect size**
		**Yes** **N=24**	**No** **N=36**		
Demographic variables	Sex(male)	16 (66.7%)	25 (69.4%)	0.52	0.029
	Age	73.25 (70.66-75.62)	68.97 (56-69.5)	0.03	1.15
Risk factors	Diabetes Mellitus	7 )29.2%(	12 )33.3%(	0.48	0.044
	Hypertension	18 (75%)	34 (94.4%)	0.03	0.270
	Hyperlipidemia	18 (75%)	23 (63.9%)	0.26	0.116
	Smoking	5) 20.8%(	9 )25%(	0.48	0.048
	Previous IHD	9 )37.5%(	20 (55.6%(	0.134	0.174
	Symptomatic	15 )62.5%(	10 )27.8%(	0.008	0.326
Anatomical variations	Lesion Calcification	11) 45.8%(	22 )58.3%(	0.18	0.150
	Vessel side (left carotid)	17 (70.8%)	17 (47.2%)	0.06	0.227
	Contralateral stenosis (%) [mean (SD) (CI)]	20.31 (35.09) (6.95-33.85)	50.0 (57.73) (0-100)	0.23	-0.59
	Ipsilateral stenosis (%) [mean SD) ( (CI)]	79.68 (35.09) (66.14-93.4)	67.50 (47.16) (17.5-100)	0.28	0.385
Procedural variations	Intraprocedure hypotension	3 )12.5%(	5 )13.9%(	0.58	0.035
	Post-procedure hypotension	16 (66.7%)	21)58.33%(	0.34	0.032
	Pre-dilation	16 )66.7%(	24 )66.7%(	0.6	0.053
	Post-dilation	4(16.7%)	8 (22.9%)	0.4	0.085
	Duration of Procedure [mean (SD)]	19.37(6.39)	17.41 (5.79)	0.22	0.44
	Stent type (hybrid(	5 )20.8%(	14 )38.9%(	0.11	0.206
Hs-CRP level	Pre-procedural [mean (SD) (CI)]	2.75 (3.06) (1.7-4.11)	2.97 (4.63) (1.7-4.63)	0.79	0.073-
	Post-procedural [mean (SD) (CI)]	9.90 (20.79) (4.8-19.64)	5.49 (7.10) (3.55-7.94)	0.06	0.42
	Post-pre difference [mean (SD) ( CI)]	6.98(19.95) (2.42-15.96)	2.52(4.29) (1.42-4.08)	0.01	0.4742

SD: standard deviation, CI: 95% confidence interval

The higher age, symptomatic lesions, negative history of hypertension and hs-CRP difference had significant association with the presence of DWI lesions in univariate analysis. Angioplasty of left carotid bulb and post-procedural hs-CRP levels was very close to the level of significance (P=0.06) (Table-2). Multivariate analysis for the abovementioned variables revealed that the risk of developing new ischemic lesions was significantly associated with left sided lesions (P=0.04), adjusted odds ratio (OR): (3.76, 95% CI: 1.08-13.13), and reversely associated with the history of hypertension) P=0.041, adjusted OR: 0.15, 95%CI: 0.02-0.93). The association between hs-CRP difference and evolution of DWI lesions remained in multivariate analysis model but it was not statistically significant (P=0.319, adjusted OR: 1.07, 95% CI: 0.94 -1.22).

## Discussion

In this prospective study, the association between hs-CRP levels and major complication of CAS was evaluated. Angioplasty was associated with higher frequency of elevated post-procedural hs-CRP in comparison to angiography. During the 30-day follow-up there was no patient with MACE. 40% of patients who underwent CAS had mircroembolic signals in post-procedural MRI. Even though the hs-CRP difference, post-procedural hs-CRP, higher age, and symptomatic lesions were associated with microembolic signals after CAS in univariate regression model, there were no significant associations in multivariate regression analysis. Left sided lesions were associated with evolution of microembolic signals and current or any previous history of hypertension had protective effect on the development of microembolic events in multivariate regression analysis. 

There was a significant association between post-procedural hs-CRP level and hs-CRP difference, and the number of new lesions. There was a significant association between the hs-CRP difference and the accumulated lesion surface area. 

Elderly patients (19, 20), symptomatic lesions (20) and left sided lesions (4) were associated with more embolic complications in previous studies. But negative association of hypertension and microeicmbolic signals, seen in current study was contrary to previous studies (19). We cannot explain this negative association since chronic hypertension is actually associated with impaired collaterals. Hs-CRP is a systemic inflammatory marker that is produced in large amount by hepatocytes in response to interleukin-1 (IL-1), interleukin-6 (IL-6) and tumor necrotic factor-α (TNF-α) after ischemic stroke (9, 21). There are several studies that have shown the diagnostic and prognostic role of hs-CRP in ischemic stroke (13).

In the present study, pre-procedural hs-CRP levels were not significantly different between patients with and without new lesions in CAS group. This finding is inconsistent with the results of Setacci et al., and Groschel et al., studies (22, 23). Groschel et al., showed that pre-procedural CRP is a predictor of stroke and death within 30 days after CAS (23). Also, Pini et al.’s study proved that the number of microembolic brain infarcts in patients with more than 5 mg/L hs-CRP concentration is significantly higher (24). 

Meanwhile, elevated hs-CRP serum concentration after CAS was associated with a higher risk of microembolic signals in univariate analysis. Several mechanisms can explain the increased risk of thromboembolic events in patients with post-procedural elevated hs-CRP. Hs-CRP aggregation in human serum can trigger the complement pathway and induce the release of inflammatory mediators (25). These inflammatory processes can directly damage the endothelial cells and disrupt its function (26). Thus, the tissue factor expression and formation of procoagulant microparticles increase and enhance the risk of clot formation (27). Hs-CRP also activates macrophages and metalloproteinase pathway, which is an indicator for presence of macrophages and T lymphocytes in arterial plaques (28). This makes the carotid plaques unstable and increases the risk of plaque rupture and microembolization. Our results were consistent with some previous studies. In Jia et al.’s study, the levels of hs-CRP, TNF-α, soluble intercellular adhesion molecule-1(sICAM-1), interleukin-8 (IL-8), fibrinogen and D-dimer had significantly increased in the CAS group, in comparison with diagnostic angiography group (29). The significant difference of hs-CRP level between CAS and diagnostic angiography in the current study can be explained by the inflammatory effects of stent implantation. Carotid angioplasty and stent deployment apply a transient retraction to the smooth muscle cells of the vessel wall that activates the inflammatory phase of vessel restoration and increases the concentration of inflammatory markers such as CRP in the serum (30, 31). While stenting, injury to intima and media layer and penetration of lipid core may induce perivascular inflammatory responses (31, 32) and since stent is a foreign object, it can trigger the immunoinflammatory responses resulting in elevated hs-CRP level. Furthermore, release of metal ions by stent material induces inflammatory reactions (33). Abe et al., showed that IL-6 and osteopontin were produced in the site of CAS (34). Moreover, CRP, IL-6 and TNF-α levels increased immediately after CAS in Xia et al., study (35). Interestingly, CRP, IL-6 and TNF-α levels were associated with number of stents. Employing the drug-eluting stents, biodegradable stents or combination of these two types (such as hybrid stents) can potentially decrease the inflammatory responses due to anti-proliferative, anti-coagulative, and anti-inflammatory effects (33). It is preferred to use less immunogenic materials and preemptive immune therapy in patients undergoing CAS (33). 

Relatively, low sample volume, short follow-up period, lack of evaluation of high density protein, and lack of evaluation of other “upstream/downstream” inflammatory biomarkers were the major shortcomings of this study. As a conclusion, the presence of some correlations between new diffusion- weighted MRI lesions and post-procedural hs-CRP with hs-CRP difference may reveal an inflammatory mechanism in the embolic complications of CAS. Utilizing drug-eluting or biodegradable stents (33), or less immunogenic stent materials (34), and preemptive immune therapy for CAS might have had therapeutic implications in the current and previously-mentioned studies. Several phase III trials were performed with drugs that had led to marked reductions in IL-6 and C-reactive protein (such as canakinumab and methotrexate for prevention of acute vascular events) (35, 36).
